# Prognostic factors for early identification of asthma: Analysis of the Korean Health Panel Survey (2014–2018)

**DOI:** 10.1097/MD.0000000000045378

**Published:** 2025-10-17

**Authors:** Seungeun Oh, Kyuhee Jo, Hyungkyun Mok

**Affiliations:** a Seoul Women’s College of Nursing, Seoul, Republic of Korea; b Department of Health Administration, Hanyang Women’s University, Seoul, Republic of Korea.

**Keywords:** asthma, Korea Health Panel, nomogram

## Abstract

This study aimed to develop and validate a predictive nomogram for asthma incidence using longitudinal panel data from South Korea. The goal was to support clinical decision-making and enhance early intervention for primary healthcare providers. Although asthma is manageable through outpatient care, its high hospitalization rate in Korea presents a significant healthcare burden. Therefore, early screening and targeted interventions are essential to improve patient outcomes. We analyzed data from 16,630 adults in the Korea Health Panel Survey (2014–2018), including 155 with asthma (J45–J46). We randomly split the data into training (70%) and validation (30%) sets. Using multivariable logistic regression, we identified significant predictors of asthma incidence. We then validated the nomogram using the concordance index (*C*-index), calibration plots, receiver operating characteristic analysis, and bootstrapping with 100 resamples. Our analysis identified male sex, age over 65, lower educational attainment, medical aid, and comorbidities as significant predictors of asthma. The model demonstrated good discriminatory power in the training set, with an area under the curve of 0.786 (95% confidence interval: 0.753–0.818) and a *C*-index of 0.798. The validated nomogram serves as a practical tool for healthcare providers to identify patients at high risk for asthma. This tool enables rapid risk assessment, facilitates targeted patient education, and supports multidisciplinary collaboration, potentially improving the quality and efficiency of asthma care.

## 1. Introduction

Asthma is a chronic respiratory system disorder involving inflammation and narrowing of the airways and it affects 262 million people.^[1]^ Asthma may cause coughing, shortness of breath, chest tightness, and wheezing.^[2]^ It is classified as a major ambulatory care-sensitive condition.^[3]^ Appropriate provision of asthma ambulatory care has been shown to reduce hospitalizations.^[4]^ In 2023, in South Korea, the utilization of pulmonary function tests, crucial for asthma diagnosis and disease management, was low, with only 24.5% of primary care clinics conducting these tests.^[5]^ Asthma management gaps stem from insufficient patient education, including poor inhaler use, the absence of a management plan, and unequal access to healthcare resources.^[6]^ As a result, in 2023, the asthma hospitalization rate in South Korea was 65 per 1,00,000 population, nearly double the Organization for Economic Cooperation and Development (OECD) average. Additionally, asthma patients accounted for 1.7% of all outpatient visits.^[7]^ Inadequate asthma management has been shown to increase the frequency of emergency department visits and hospitalizations.^[8]^ The prevalence of asthma among South Korean adults aged 19 and older increased from 2.3% in 2017 to 3.1% in 2023, highlighting its status as a significant public health concern.^[9]^ The rising prevalence and inadequate management of asthma are reducing patients’ quality of life and increasing healthcare utilization.^[10]^

Asthma is one of the preventable chronic diseases.^[11]^ One of the main ways to prevent asthma is to avoid its associated risk factors.^[12]^ Several factors are known to be involved in the genetics, environment, lifestyle, and socioeconomics that lead to asthma.^[13–15]^ Identifying and understanding these risk factors is crucial for prevention and treatment plans.^[16]^ This requires asthmatic patient education and management by primary healthcare providers.^[17]^ Primary healthcare providers play a key role in effectively managing these risk factors through patient education, asthma management plan development, and ongoing monitoring.^[18]^

In 2021, the socioeconomic burden of asthma in South Korea was estimated to be $2.7 billion, which shows an upward trend.^[19]^ This financial impact underscores the need for targeted interventions and policies aimed at reducing both the prevalence and the economic burden of asthma, particularly in vulnerable communities.^[20]^ Socioeconomic status is associated with asthma, where the prevalence of asthma is higher in groups of lower status.^[21]^ Additionally, access to healthcare and education about asthma management can significantly influence outcomes, highlighting the importance of addressing these disparities to improve overall health in affected populations.^[22]^ The identification and early intervention of factors contributing to the development of asthma is of critical importance^[23]^ and is a key area of responsibility for primary healthcare providers.^[24]^ Early detection is critical to the management and prevention of asthma exacerbations,^[25]^ and this requires a multifaceted approach that includes primary healthcare providers-led public awareness campaigns, improved access to healthcare resources, and community support initiatives.^[26]^

While existing asthma prediction tools have limitations,^[27]^ nomograms offer a practical and intuitive alternative for rapid risk assessment.^[28,29]^ They can help primary care providers identify prognostic indicators and facilitate early detection, thereby reducing the national healthcare burden.^[30]^ However, a nomogram for asthma incidence based on longitudinal Korean data has not yet been developed. To address this gap, this study aimed to analyze the Korean Health Panel data (2014–2018) to identify factors associated with asthma. Our ultimate objective was to develop and validate a predictive nomogram that provides a practical tool for personalized risk assessment and intervention in clinical practice.

## 2. Materials and methods

### 2.1. Data source

The data used in this study were obtained from the Korea Health Panel Survey (KHPS), a nationally representative secondary data collection conducted annually by the Korea Institute for Health and Social Affairs (KIHASA) and the National Health Insurance Service (NHIS). The KHPS utilizes a stratified cluster sampling method based on population and housing census, where a class is an administrative district, and a cluster is a household. Family members from selected households participate in the KHPS survey, which examines demographic and socioeconomic characteristics, healthcare service use, medical expenditure, and access to care. The KHP data yearly followed up nationally representative 6640 households (18,049 individuals) since 2008. The survey included a self-reported questionnaire and in-person interviews with well-trained investigators. All references to the usage of healthcare services were clarified by expense tracker, medical bill, tax filing documents, or medical document of each individual, including the Korean Standard Classification of Diseases, 7th Revision.^[31]^ The purpose of the KHPS is to provide representative information on the use of medical services by the Korean population and to develop policies that improve the efficiency and accessibility of the Korean healthcare system.

### 2.2. Study population

**Figure 1.**
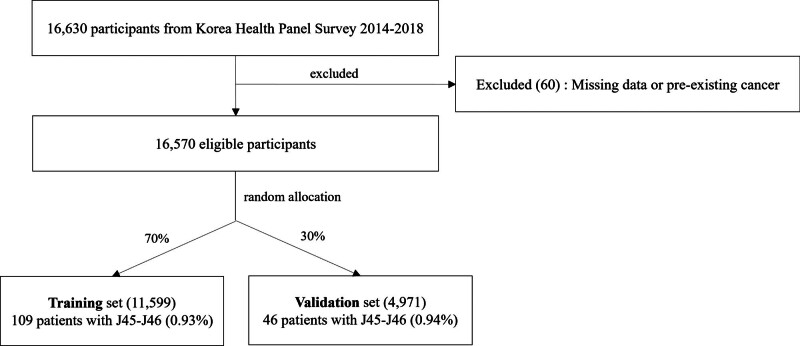
Study flow chart.

The study population for this study was selected based on the purpose of the study using the KHPS data from 2014 to 2018. This pre-COVID-19 pandemic timeframe provides a stable baseline for asthma incidence and healthcare utilization patterns, unaffected by the significant disruptions to the healthcare system and public health behaviors caused by the pandemic. To determine the total number of adults aged over 18 years, all subjects were included in the initial data for each year. We excluded the 60 subjects who did not answer the question or had preexisting cancer (all types) were excluded. To identify people with asthma, we considered the International Classification of Diseases (ICD-10) based on the assigned Korean Standard Classification of Diseases, 7th Revision, following the guidelines of the KHPS. In the KHPS data, respondents with asthma diagnosis codes J45 and J46 were classified as having asthma. The selection process of the study population is illustrated in the study flow chart (Fig. 1).

### 2.3. Covariates

Ten variables were adjusted for in this study, including sex, age, education level, household income, marital status, national health insurance program, body mass index (BMI), Charlson comorbidity index (CCI) score, pack-years, and quality of life. Age was divided into 3 groups: 19 to 44 years old, 45 to 64 years old, and 65 years old or older, and education level was categorized as high school diploma or lower and college degree or higher. Since household income is the income of the household unit, it was adjusted by dividing it by the square root of the number of household members and then classified into 5 quintile groups. Marital status was classified as married, single, divorced, or widowed. Insurance types were divided into national health insurance and medical aid. A BMI of 25 or higher was considered as obesity.^[32]^ The CCI is a method of adjusting the sum by assigning weights of 1 to 6 points based on the severity of 17 disease groups. Weighted scores were assigned by converting to ICD-10 codes corresponding to the 17 disease groups of CCI, and this study analyzed it by dividing it into “0, 1, 2+.” Smoking history was measured in terms of pack-years, which indicates the number of years of smoking, in units of 20 cigarettes per pack, and was calculated using the current/past smoking amount variable. Pack-years were calculated as “number of packs of cigarettes smoked per day × total number of years of smoking” and were classified based on 10 years. The EQ-5D, a tool to measure health-related quality of life, was used to measure quality of life. The EQ-5D questions were divided into 5 dimensions: mobility, self-care, everyday activities, pain/discomfort, and anxiety/depression, and were organized and classified into 3 scales (no difficulty, some difficulty, and a lot of difficulty). The closer the score is to 1, the better the health status.

### 2.4. Statistical analysis

We randomly split the data into a training set (n = 11,599) and a validation set (n = 4971) in a 7:3 ratio. Quantitative variables were expressed as mean ± standard deviation (Mean ± SD), and a *t*-test was used to compare between groups. Qualitative variables were expressed as the number and percentage, and a χ^2^ test was used for comparison. Univariate and multivariable logistic regressions were used to identify independent predictors of asthma. For the multivariable model, variables from the univariate analysis with a *P*-value < .20 were included. To assess for multicollinearity among the predictors, we calculated the variance inflation factor for each variable in the final model. All variance inflation factor values were below 2.0, indicating that multicollinearity was not a significant concern. Although the overall incidence of asthma in our cohort was low, we assessed the adequacy of our sample size for this model using the events per variable (EPV) criterion. Our final model included 8 predictor variables. With 155 asthma events, this results in an EPV of 19.4 (155 events/8 variables). This value is substantially higher than the commonly accepted minimum threshold of 10, suggesting that the sample size was sufficient to avoid overfitting and ensure the stability of the regression coefficients.^[33]^ Data from both the training and validation sets were used to develop and internally validate a nomogram. The performance of the nomogram was assessed using the concordance index (*C*-index), receiver operating characteristic (ROC) curves, and calibration plots with 100 bootstrap resamples to minimize overfitting. The *C*-index was used to evaluate the ability to differentiate between various outcomes, with a higher *C*-index value indicative of superior model performance. Calibration plots were utilized to measure the agreement between predicted and observed probabilities. All statistical analyses were performed using R software (version 4.4.2, R Foundation, https://www.r-project.org/), and a *P*-value of <.05 was considered statistically significant.

### 2.5. Ethical consideration

This study was reviewed and approved by the Institutional Review Board of the Korea National Institute for Bioethics Policy (IRB No. P01-202211-01-028). The approval included the study protocol, data handling procedures, and research ethics pledge.

## 3. Results

### 3.1. General characteristics

The study population consisted of a total of 16,570 subjects, including 7866 males (47.5%) and 8704 females (52.5%). The subjects were divided into a training set (n = 11,599) and a validation set (n = 4971). In the training set, 4123 (35.6%) were aged 45 to 64, 6892 (59.4%) were high school diploma or lower, 8894 (76.7%) had a CCI score of 1, 387 (3.3%) were medical aid recipients, and 109 (0.9%) had asthma. There were no statistically significant differences between any of the variables in the training and validation sets (all *P* > .05; Table 1).

**Table 1:** Demographic and clinical characteristics of the study cohort.

Variable	Training set (n = 11,599)		Validation set (n = 4971)		Overall (n = 16570)		*t*/χ^2^	*P*-value
	N	%	N	%	N	%		
Sex								
Male	5532	47.7	2334	47.0	7866	47.5	0.767	.381
Female	6067	52.3	2637	53.0	8704	52.5		
Age (yr)								
19–44	3796	32.7	1646	33.1	5442	32.8	5.395	.067
45–64	4123	35.6	1835	36.9	5958	36.0		
≥65	3680	31.7	1490	30.0	5170	31.2		
Education level								
High school diploma or lower	6892	59.4	2940	59.1	9832	59.3	0.110	.740
College degree or higher	4707	40.6	2031	40.9	6738	40.7		
Household income (quintiles)								
Q1 (lowest 20%)	1601	13.7	666	13.4	2267	13.7	1.418	.841
Q2	2069	17.7	873	17.6	2942	17.7		
Q3	2369	20.4	1008	20.3	3377	20.4		
Q4	2696	23.5	1192	24.0	3888	23.5		
Q5 (highest 20%)	2864	24.7	1232	24.7	4096	24.7		
Marital status								
Married	7319	63.1	3138	63.1	10,457	63.1	0.511	775
Single	2618	22.6	1139	22.9	3757	22.7		
Divorced, widowed, separated, missing	1662	14.3	694	14.0	2356	14.2		
National health insurance program								
NHI	11,212	96.7	4795	96.5	16,007	96.6	0.441	.506
Medical aid	387	3.3	176	3.5	563	3.4		
BMI								
<25	8807	75.9	3754	75.5	12,561	75.8	0.320	.571
≥25	2792	24.1	1217	24.5	4009	24.2		
CCI score								
0	1791	15.4	750	15.1	2541	15.3	1.160	.560
1	8894	76.7	3848	77.4	12,742	76.9		
2+	914	7.9	373	7.5	1287	7.8		
Pack-years								
<10	7719	66.5	3367	67.7	11,086	66.9	2.203	.138
≥10	3880	33.5	1604	32.3	5484	33.1		
Quality of life	0.943 ± 0.861		0.944 ± 0.869				0.720	.472
Asthma								
No	11,490	99.1	4925	99.1	16,415	99.1	0.008	.930
Yes	109	0.9	46	0.9	155	0.9		

BMI = body mass index, CCI = Charlson comorbidity index, NHI = national health insurance.

### 3.2. Demographics and clinical characteristics comparison by asthma groups

In the training set, the variables of sex, BMI, and pack-years were not statistically different between the asthmatic and non-asthmatic groups (*P* > .05). Among those aged 65 years or older, 85 (78.0%) were diagnosed with asthma, while non-asthma was present in >30% of all age groups. Within the asthma group, 99 (90.8%) were high school diploma or lower, 13 (11.9%) were on medical aid, and 23 (21.1%) had a CCI score of 2 or higher, which was higher than that of the non-asthmatic group (Table 2).

**Table 2:** Demographic and clinical characteristics by non-asthma and asthma groups in training set.

Variable	Non-asthma (n = 11,490)		Asthma (n = 109)		Overall (n = 11599)		*t*/χ^2^	*P*-value
	N	%	N	%	N	%		
Sex								
Male	5478	47.7	54	49.5	5532	47.7	0.151	.698
Female	6012	52.3	55	50.5	6067	52.3		
Age (yr)								
19–44	3790	33.0	6	5.5	3796	32.8	110.330	<.001
45–64	4105	35.7	18	16.5	4123	35.5		
≥65	3595	31.3	85	78.0	3680	31.7		
Education level								
High school diploma or lower	6793	59.1	99	90.8	6892	59.4	45.012	<.001
College degree or higher	4697	40.9	10	9.2	4707	40.6		
Household income (quintiles)								
Q1 (lowest 20%)	1560	13.6	41	37.6	1601	13.8	64.983	<.001
Q2	2042	17.8	27	24.8	2069	17.8		
Q3	2352	20.5	17	15.6	2369	20.4		
Q4	2683	23.4	13	11.9	2696	23.2		
Q5 (highest 20%)	2853	24.7	11	10.1	2864	24.8		
Marital status								
Married	7251	63.1	68	62.4	7319	63.1	51.535	<.001
Single	2615	22.8	3	2.8	2618	22.6		
Divorced, widowed, separated, missing	1624	14.1	38	34.8	1662	14.3		
National health insurance program								
NHI	11,116	96.7	96	88.1	11,212	96.7	25.175	<.001
Medical aid	374	3.3	13	11.9	387	3.3		
BMI								
<25	8726	75.9	81	74.3	8807	75.9	0.157	.692
≥25	2764	24.1	28	25.7	2792	24.1		
CCI score								
0	1786	15.5	5	4.6	1791	15.4	32.883	<.001
1	8813	76.7	81	74.3	8894	76.7		
2+	891	7.8	23	21.1	914	7.9		
Pack-years								
<10	7653	66.6	66	60.6	7719	66.5	1.778	.182
≥10	3837	33.4	43	39.4	3880	33.5		
Quality of life	0.944 ± 0.857		0.888 ± 0.103				6.676	<.001

BMI = body mass index, CCI = Charlson comorbidity index, NHI = national health insurance.

### 3.3. Predictors of asthma

In the logistic regression analysis, age (19–44 reference group vs ≥65: univariate odds ratio (OR): 14.935 [6.518–34.222], multivariable OR: 3.803 [1.306–11.072]), education level (college degree or higher reference group vs high school diploma or lower: univariate OR: 6.845 [3.569–13.131], multivariable OR: 2.286 [1.092–4.784]), national health insurance program (NHI reference group vs medical aid: univariate OR: 4.025 [2.235–7.249], multivariable OR: 1.924 (1.000–3.703]), and CCI score (0 reference group vs 2+: univariate OR: 9.221 [3.494–24.335], multivariable OR: 4.344 [1.635–11.539]) were associated with asthma. While sex did not demonstrate significance in the univariate analysis, it was found to be significant in the multivariable analysis (female reference group vs male: univariate OR: 1.078 [0.739–1.571], multivariable OR: 1.624 [1.064–2.478]). Conversely, household income and marital status were significant in the univariate analysis, but they did not show significance in the multivariable analysis (Q5 [highest 20%] reference group vs Q1 [lowest 20%]: univariate OR: 6.817 [3.494–13.300], multivariable OR: 1.426 [0.666–3.053]; Table 3).

**Table 3:** Multivariable logistic regression in the training set.

	Univariate		Multivariable	
	OR (95% CI)	*P*-value	OR (95% CI)	*P*-value
Sex				
Female (ref)				
Male	1.078 (0.739–1.571)	.698	1.624 (1.064–2.478)	.025
Age				
19–44 (ref)				
45–64	2.770 (1.098–6.985)	.031	1.214 (0.415–3.550)	.723
≥65	14.935 (6.518–34.222)	<.001	3.803 (1.306–11.072)	.014
Education level				
College degree or higher (ref)				
High school diploma or lower	6.845 (3.569–13.131)	<.001	2.286 (1.092–4.784)	.028
Household income (quintiles)				
Q5 (highest 20%; ref)				
Q4	1.257 (0.562–2.810)	.578	0.923 (0.408–2.086)	.847
Q3	1.875 (0.876–4.010)	.105	1.036 (0.474–2.264)	.929
Q2	3.429 (1.697–6.929)	.001	1.178 (0.555–2.497)	.670
Q1 (lowest 20%)	6.817 (3.494–13.300)	<.001	1.426 (0.666–3.053)	.361
Marital status				
Married (ref)				
Single	0.122 (0.038–0.389)	<.001	0.401 (0.102–1.574)	.190
Divorced, widowed, separated, missing	2.495 (1.672–3.724)	<.001	1.363 (0.856–2.168)	.192
National health insurance program				
NHI (ref)				
Medical aid	4.025 (2.235–7.249)	<.001	1.924 (1.000–3.703)	.050
BMI				
<25 (ref)				
≥25	1.091 (0.709–1.681)	.692	1.001 (0.647–1.549)	.996
CCI score				
0 (ref)				
1	3.283 (1.329–8.112)	.010	3.237 (1.297–8.083)	.012
2+	9.221 (3.494–24.335)	<.001	4.344 (1.635–11.539)	.003
Pack-years				
<10 (ref)				
≥10	1.299 (0.883–1.912)	.184	1.192 (0.804–1.768)	.382
Quality of life	0.032 (0.011–0.095)	<.001	0.345 (0.061–1.950)	.228

BMI = body mass index, CI = condidence interval, CCI = Charlson comorbidity index, NHI = national health insurance, OR =odds ratio.

### 3.4. Nomogram predicting the probability of developing asthma

**Figure 2.**
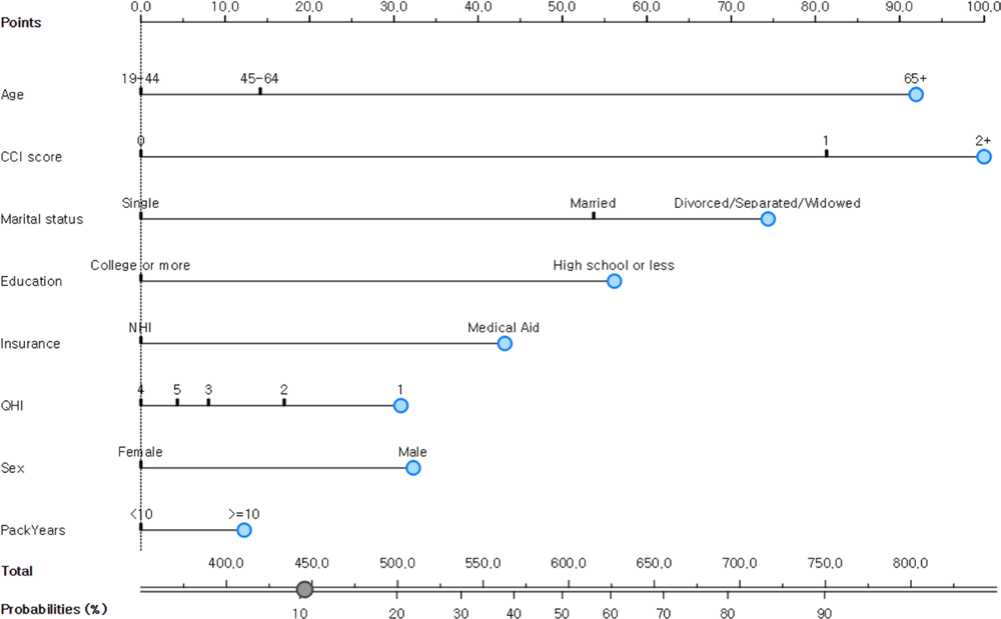
Nomogram for predicting asthma. CCI = Charlson comorbidity index, NHI = national health insurance, QHI = quality of health life.

Using the results from our multivariable logistic regression, we developed a predictive nomogram to estimate the incidence of asthma. This visual aid incorporates key predictive factors: CCI score, age, marital status, education level, insurance, sex, household income, and pack-years (Fig. 2). This visual predictor is a practical tool for healthcare providers. To use the nomogram, 1 identifies a patient’s value for each predictor on its corresponding axis, draws a vertical line up to the “Points” axis to determine the score for that variable, and sums the scores for all variables to get a “Total Points” score. A vertical line is then drawn down from the “Total Points” axis to the “Predicted Value” axis to find the individual’s probability of asthma incidence. For instance, a 67-year-old male (age ≥ 65, 92 points) who is divorced (32 points), has a high school diploma (education, 74 points), receives medical aid (insurance, 56 points), and has a CCI score of 2 or higher (100 points) would have a total score of 354 points. This score corresponds to a predicted asthma probability of approximately 10%, justifying the need for targeted preventive measures.

**Figure 3.**
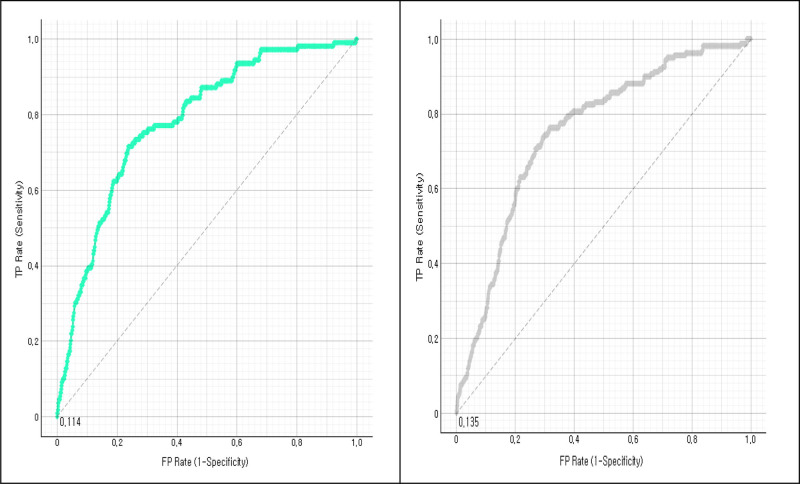
ROC curves of the training set and the validation set (Training set = mint, Validation = gray). FP = false positive, ROC = receiver operating characteristic, TP = true positive.

**Figure 4.**
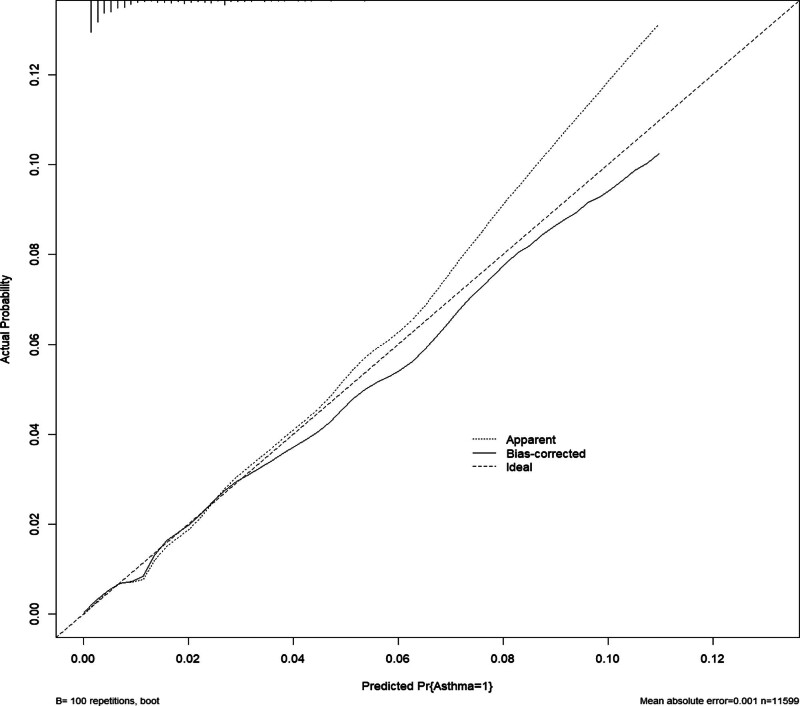
Calibration curve for the nomogram by training set.

We evaluated the nomogram’s efficacy using ROC curve and calibration curve analysis. The area under the curve (AUC) values were derived from the outcomes of the ROC analysis. The training dataset produced an AUC value of 0.786 (95% confidence interval [CI]: 0.753–0.818). Similarly, the validation set had an AUC value of 0.777 (95% CI: 0.725–0.829). The AUC values in Figure 3 indicated robust discriminatory power. The concordance index (*C*-index) in the training set was calculated at 0.798 (95% CI: 0.761–0.834), demonstrating a high level of agreement between the predicted probabilities and observed outcomes. The calibration curve revealed a close correspondence between predicted and actual probabilities, indicating predictive solid accuracy (Fig. 4).

To evaluate the overall performance of the nomogram, we calculated the Brier score, which measures the mean squared difference between predicted probabilities and actual outcomes. A lower Brier score indicates better model performance. The Brier score for our model was 0.0093 in the training set and 0.0092 in the validation set. To place this value in context, we compared it to the Brier score of a null model. A null model serves as a baseline for performance by making the simplest possible prediction. It assigns the overall incidence rate of the outcome (0.9% in our cohort) to every individual, without using any predictor variables. This null model yields a Brier score of approximately 0.0094. Therefore, our model’s lower Brier score, while modest, demonstrates a clear improvement in predictive accuracy over this baseline and confirms its clinical utility.

## 4. Discussion

The persistently high prevalence of asthma, coupled with hospitalization rates double the Organization for Economic Co-operation and Development (OECD) average, establishes asthma as a major public health concern in South Korea.^[34,35]^ Addressing this challenge requires a deeper understanding of its risk factors to alleviate the national healthcare burden. In this context, our study provides valuable insights by developing a predictive nomogram based on key demographic, socioeconomic, and clinical predictors. The following discussion will elaborate on these findings and their clinical implications.

We found that sex was significantly associated with asthma. While we analyzed that sex did not have a significant association with asthma incidence in our univariate analysis, after controlling for socioeconomic factors, the CCI, pack-years of smoking, and quality of life variables, we found that males were more likely to have asthma than females. According to the previous studies, the association between sex and asthma that we found varies by age group. Up to the age of 50, female may be at a higher risk of having asthma than male. However, after the age of 50, the difference in asthma incidence between sex tends to decrease, with male and female experiencing similar rates of asthma.^[36]^ Occupational exposure to steam, dust, and smog can worsen asthma, and the condition has long been thought to have a sex difference in prevalence varying across countries and industries. For example, in Germany, male are typically more affected by asthma than female in the bakery and lumber industries. In contrast, female have a higher prevalence of asthma in the beauty industry.^[37]^ Our findings indicated that after controlling the various risk factors, individuals aged 65 and older were about 4 times as likely to have asthma compared to those under the age of 45. The possibility of having asthma began to increase starting at age 45, and the mortality rate from asthma for individuals age 55 and older was significantly higher than that of younger adults.^[38,39]^ Poor educational attainment has also been associated with a higher incidence of asthma.^[40]^ According to our study, respondents with a bachelor’s degree or higher had a lower incidence of asthma than those without a bachelor’s degree. An extensive investigation into the educational backgrounds of young adults in 4 US cities also revealed a strong correlation between low education and high asthma rates.^[41]^ Poorly educated people might be less able to navigate the healthcare system, and their health literacy might need to be improved. This appears especially relevant for people living with asthma since 1 investigation found that Chicago adults with long-term asthma and low health literacy were much more likely to make emergency room visits than those with asthma with high health literacy.^[42]^ Low education levels may also cause income inequality and are a risk factor for asthma.^[43]^ Moreover, fewer educational opportunities frequently accompany problems in obtaining health insurance since job opportunities are not only limited but also frequently come without the benefit of insurance. Whether poor education leads to the health-damaging condition known as asthma, 1 thing is clear: low social and economic factors and worsened health are linked.^[44]^ In the United States, adults with lower levels of education experience higher unemployment rates than those with more advanced education.^[45]^ The absence of employment-related benefits, such as employer-sponsored health insurance, exacerbates disparities in healthcare access.^[46]^ Failure to enroll in health insurance increases the risk of declining health, leading to increased emergency room visits and worsening conditions like asthma.^[47]^ Our study found that individuals without national health insurance were more likely to suffer from asthma. We found an association between comorbidities and asthma incidence. The elderly with comorbidities are more likely to have asthma than younger adults.^[48]^ The correlation between aging and comorbidities is well known. The aging of the world population is leading to a steady increase in the proportion of elderly individuals, increasing the socioeconomic burden of chronic diseases.^[49]^ As patients age, the likelihood of developing other diseases increases, making it crucial to manage comorbidities in elderly patients.^[50]^ In this study’s nomogram, the highest probability of having asthma was observed in elderly with a CCI score of 2 or higher. This study’s multivariable logistic regression analysis showed that asthma and previously statistically insignificant variables, such as marital status and quality of life, also impacted the nomogram prediction probability. Other studies have also found a correlation between these risk factors and asthma. For example, a Finnish longitudinal study of adults without asthma symptoms found that individuals who experienced interpersonal conflicts due to divorce or separation had a 1.7 times higher risk of developing asthma.^[51]^ Similarly, longitudinal studies conducted across all age groups in Sweden and Norway have found an association between low quality of life and the onset of asthma.^[52]^

The clinical utility of this nomogram lies in its ability to translate a statistical probability into actionable clinical guidance. An AUC of 0.786 indicates a good ability to distinguish between individuals who will and will not develop asthma. In a primary care setting, clinicians could establish a risk threshold to guide interventions. For instance, a predicted asthma risk of ≥10%, as illustrated in our example, could trigger a recommendation for targeted preventive education on environmental triggers and lifestyle modifications. A lower threshold might prompt more frequent follow-ups, allowing for a stratified approach to care that focuses resources on patients at higher risk.

The nomogram developed in this study has the potential to serve as a practical tool to support primary healthcare providers’ decision-making processes and enhance their effectiveness in clinical settings. It is anticipated to be particularly beneficial for the rapid screening of asthma risk groups and the implementation of early interventions in settings where primary healthcare providers interact directly with patients, such as primary care centers, public health centers, and school health centers. The utilization of nomograms by primary healthcare providers is expected to facilitate a systematic assessment of patients’ risk factors, including smoking, comorbidity, and socioeconomic status. This, in turn, should enable the provision of tailored health education and lifestyle modification programs, thereby enhancing patient outcomes.

In order to optimize the effectiveness of asthma management, a multidisciplinary approach using nomograms is required. Asthma management teams comprising physicians, nurses, respiratory therapists, pharmacists, and other healthcare professionals can share nomogram results and plan integrated interventions based on their expertise. Furthermore, the integration of nomograms into health policies is expected to enhance healthcare quality through the standardization of patient risk screening and management.

Our nomogram contributes to the existing literature on asthma prediction. While early models focused on limited clinical variables, more recent studies in the last 3 years have explored machine learning algorithms using electronic health records.^[53,54]^ These models often achieve high predictive accuracy but require significant computational resources and data integration, limiting their use in primary care settings. In contrast, our nomogram is a simple, transparent tool based on easily obtainable patient characteristics, making it highly applicable for rapid risk screening at the point of care.

### 4.1. Limitations

This study has several limitations that should be acknowledged. First, the reliance on self-reported data for variables like smoking history and quality of life is subject to recall bias. Second, although the EPV criterion was met, the number of asthma cases (n = 155) remains relatively small, which warrants caution. Third, our model was validated internally using bootstrapping. While this method provides a robust assessment of the model’s performance within our dataset, external validation in an independent cohort is necessary to confirm its generalizability and transportability to other populations. Therefore, future studies should aim to validate this nomogram in diverse clinical settings.

## 5. Conclusion

This study analyzed national data from the Korea Health Panel Study (2014–2018) to identify key risk factors for asthma in South Korean adults. Based on these findings, we developed a practical nomogram for clinical application. The predictive model identified several significant risk factors: male sex, age 65 and older, lower educational attainment, medical aid status, and the presence of comorbidities. The resulting nomogram is a practical tool designed to enhance clinical decision-making in primary care settings, public health centers, and school health offices. It enables healthcare providers to conduct rapid risk assessments, provide tailored patient education, and implement timely interventions for asthma prevention and management. Furthermore, integrating this nomogram into healthcare policy could standardize risk screening and facilitate multidisciplinary collaboration. Ultimately, these findings contribute not only to reducing the healthcare burden but also to enabling more precise, patient-centered management strategies for individuals at risk of asthma.

## Author contributions

**Conceptualization:** Seungeun Oh.

**Data curation:** Hyungkyun Mok.

**Formal analysis:** Seungeun Oh.

**Methodology:** Kyuhee Jo.

**Writing – original draft:** Seungeun Oh.

**Writing – review & editing:** Hyungkyun Mok.
